# Cannabidiol as a Promising Strategy to Treat and Prevent Movement Disorders?

**DOI:** 10.3389/fphar.2018.00482

**Published:** 2018-05-11

**Authors:** Fernanda F. Peres, Alvaro C. Lima, Jaime E. C. Hallak, José A. Crippa, Regina H. Silva, Vanessa C. Abílio

**Affiliations:** ^1^Laboratory of Behavioral Neurosciences, Department of Pharmacology, Federal University of São Paulo, São Paulo, Brazil; ^2^National Institute for Translational Medicine (INCT-TM, CNPq, FAPESP, CAPES), Ribeirão Preto, Brazil; ^3^Department of Neuroscience and Behavior, University of São Paulo, Ribeirão Preto, Brazil

**Keywords:** cannabidiol, movement disorders, Parkinson's disease, Huntington's disease, dystonic disorders, cannabinoids

## Abstract

Movement disorders such as Parkinson's disease and dyskinesia are highly debilitating conditions linked to oxidative stress and neurodegeneration. When available, the pharmacological therapies for these disorders are still mainly symptomatic, do not benefit all patients and induce severe side effects. Cannabidiol is a non-psychotomimetic compound from *Cannabis sativa* that presents antipsychotic, anxiolytic, anti-inflammatory, and neuroprotective effects. Although the studies that investigate the effects of this compound on movement disorders are surprisingly few, cannabidiol emerges as a promising compound to treat and/or prevent them. Here, we review these clinical and pre-clinical studies and draw attention to the potential of cannabidiol in this field.

## Cannabidiol (CBD)

Cannabidiol (CBD) is one of the over 100 phytocannabinoids identified in *Cannabis sativa* (ElSohly and Gul, 2014), and constitutes up to 40% of the plant's extract, being the second most abundant component (Grlic, 1976). CBD was first isolated from marijuana in 1940 by Adams et al. (1940) and its structure was elucidated in 1963 by Mechoulam and Shvo (1963). Ten years later, Perez-Reyes et al. (1973) reported that, unlike the main constituent of cannabis Δ^9^-tetrahydrocannabinol (Δ^9^-THC), CBD does not induce psychological effects, leading to the suggestion that CBD was an inactive drug. Nonetheless, subsequent studies demonstrated that CBD modulates the effects of Δ^9^-THC and displays multiple actions in the central nervous system, including antiepileptic, anxiolytic and antipsychotic effects (Zuardi, 2008).

Interestingly, CBD does not induce the cannabinoid tetrad, namely hypomotility, catalepsy, hypothermia, and antinociception. In fact, CBD mitigates the cataleptic effect of Δ^9^-THC (El-Alfy et al., 2010). Clinical and pre-clinical studies have pointed to beneficial effects of CBD on the treatment of movement disorders. The first studies investigated CBD's actions on dystonia, with encouraging results. More recently, the studies have been focusing on Parkinson's (PD) and Huntington's (HD) diseases. The mechanisms whereby CBD exerts its effects are still not completely understood, mainly because several targets have been identified. Of note, CBD displays anti-inflammatory and antioxidant actions (Campos et al., 2016), and both inflammation and oxidative stress are linked to the pathogenesis of various movement disorders, such as PD (Farooqui and Farooqui, 2011; Niranjan, 2014), HD (Sánchez-López et al., 2012), and tardive dyskinesia (Zhang et al., 2007).

It is noteworthy that, when available, the pharmacological treatments for these movement disorders are mainly symptomatic and induce significant side effects (Connolly and Lang, 2014; Lerner et al., 2015; Dickey and La Spada, 2017). Nonetheless, despite its great clinical relevance, the studies evaluating CBD's role on the pharmacotherapy of movement disorders are surprisingly few. Here, we will review the clinical and pre-clinical evidence and draw attention to the potential of CBD in this field.

## CBD's mechanisms of action

CBD has several molecular targets, and new ones are currently being uncovered. CBD antagonizes the action of CB_1_ and CB_2_ receptors agonists, and is suggested to act as an inverse agonist of these receptors (Pertwee, 2008). Moreover, recent evidence point to CBD as a non-competitive negative allosteric modulator of CB_1_ and CB_2_ (Laprairie et al., 2015; Martínez-Pinilla et al., 2017). CBD is also an agonist of the vanilloid receptor TRPV1 (Bisogno et al., 2001), and the previous administration of a TRPV1 antagonist blocks some of CBD effects (Long et al., 2006; Hassan et al., 2014). In parallel, CBD inhibits the enzymatic hydrolysis and the uptake of the main endocannabinoid anandamide (Bisogno et al., 2001), an agonist of CB_1_, CB_2_ and TRPV1 receptors (Pertwee and Ross, 2002; Ross, 2003). The increase in anandamide levels induced by CBD seems to mediate some of its effects (Leweke et al., 2012). Moreover, in some behavioral paradigms the administration of an inhibitor of anandamide metabolism promotes effects similar to CBD (Pedrazzi et al., 2015; Stern et al., 2017).

CBD has also been shown to facilitate the neurotransmission mediated by the serotonin receptor 5-HT_1A_. It was initially suggested that CBD would act as an agonist of 5-HT_1A_ (Russo et al., 2005), but the latest reports propose that this interaction might be allosteric or through an indirect mechanism (Rock et al., 2012). Although this interaction is not fully elucidated, multiple CBD's effects were reported to depend on 5-HT_1A_ activation (Espejo-Porras et al., 2013; Gomes et al., 2013; Pazos et al., 2013; Hind et al., 2016; Sartim et al., 2016; Lee et al., 2017).

The peroxisome proliferator-activated receptor γ (PPARγ) is a nuclear receptor involved in glucose metabolism and lipid storage, and PPARγ ligands have been reported to display anti-inflammatory actions (O'Sullivan et al., 2009). Data show that CBD can activate this receptor (O'Sullivan et al., 2009), and some of CBD effects are blocked by PPARγ antagonists (Esposito et al., 2011; Dos-Santos-Pereira et al., 2016; Hind et al., 2016). CBD also up-regulates PPARγ in a mice model of multiple sclerosis, an effect suggested to mediate the CBD's anti-inflammatory actions (Giacoppo et al., 2017b). In a rat model of Alzheimer's disease, CBD, through interaction with PPARγ, stimulates hippocampal neurogenesis, inhibits reactive gliosis, induces a decline in pro-inflammatory molecules, and consequently inhibits neurodegeneration (Esposito et al., 2011). Moreover, in an *in vitro* model of the blood-brain barrier, CBD reduces the ischemia-induced increased permeability and VCAM-1 levels—both effects are attenuated by PPARγ antagonism (Hind et al., 2016).

CBD also antagonizes the G-protein-coupled receptor GPR55 (Ryberg et al., 2007). GPR55 has been suggested as a novel cannabinoid receptor (Ryberg et al., 2007), but this classification is controversial (Ross, 2009). Currently, the phospholipid lysophosphatidylinositol (LPI) is considered the GPR55 endogenous ligand (Morales and Reggio, 2017). Although only few studies link the CBD effect to its action on GPR55 (Kaplan et al., 2017), it is noteworthy that GPR55 has been associated with PD in an animal model (Celorrio et al., 2017) and with axon growth *in vitro* (Cherif et al., 2015).

More recently, CBD was reported to act as inverse agonist of the G-protein-coupled orphan receptors GPR3, GPR6, and GPR12 (Brown et al., 2017; Laun and Song, 2017). GPR6 has been implicated in both HD and PD. Concerning animal models of PD, GPR6 deficiency was related to both diminished dyskinesia after 6-OHDA lesion (Oeckl et al., 2014), and increased sensitivity to MPTP neurotoxicity (Oeckl and Ferger, 2016). Moreover, Hodges et al. (2006) described decreased expression of GPR6 in brain of HD patients, compared to control. GPR3 is suggested as a biomarker for the prognosis of multiple sclerosis (Hecker et al., 2011). In addition, GPR3, GPR6, and GPR12 have been implicated in cell survival and neurite outgrow (Morales et al., 2018).

CBD has also been reported to act on mitochondria. Chronic and acute CBD administration increases the activity of mitochondrial complexes (I, II, II-III, and IV), and of creatine kinase in the brain of rats (Valvassori et al., 2013). In a rodent model of iron overload—that induces pathological changes that resemble neurodegenerative disorders—CBD reverses the iron-induced epigenetic modification of mitochondrial DNA and the reduction of succinate dehydrogenase's activity (da Silva et al., 2018). Of note, multiple studies associate mitochondrial dysfunctions with the pathophysiology of PD (Ammal Kaidery and Thomas, 2018).

In parallel, several studies show anti-inflammatory and antioxidant actions of CBD (Campos et al., 2016). CBD treatment decreases the levels of the pro-inflammatory cytokines IL-1β, TNF-α, IFN-β, IFN-γ, IL-17, and IL-6 (Watzl et al., 1991; Weiss et al., 2006; Esposito et al., 2007, 2011; Kozela et al., 2010; Chen et al., 2016; Rajan et al., 2016; Giacoppo et al., 2017b), and increases the levels of the anti-inflammatory cytokines IL-4 and IL-10 (Weiss et al., 2006; Rajan et al., 2016). In addition, it inhibits the expression of iNOS (Esposito et al., 2007; Pan et al., 2009; Chen et al., 2016; Rajan et al., 2016) and COX-2 (Chen et al., 2016) induced by distinct mechanisms. CBD also displays antioxidant properties, being able to donate electrons under a variable voltage potential and to prevent the hydroperoxide-induced oxidative damage (Hampson et al., 1998). In rodent models of PD and HD, CBD up-regulates the mRNA levels of the antioxidant enzyme superoxide dismutase (Garcia-Arencibia et al., 2007; Sagredo et al., 2007). In accordance, CBD decreases oxidative parameters in *in vitro* models of neurotoxicity (Hampson et al., 1998; Iuvone et al., 2004; Mecha et al., 2012). Of note, the anti-inflammatory and antioxidant effects of CBD on lipopolysaccharide-stimulated murine macrophages are suppressed by a TRPV1 antagonist (Rajan et al., 2016). It has also been shown that CBD can affect the expression of several genes involved in zinc homeostasis, which is suggested to be linked to its anti-inflammatory and antioxidant actions (Juknat et al., 2012).

**Figure 1:**
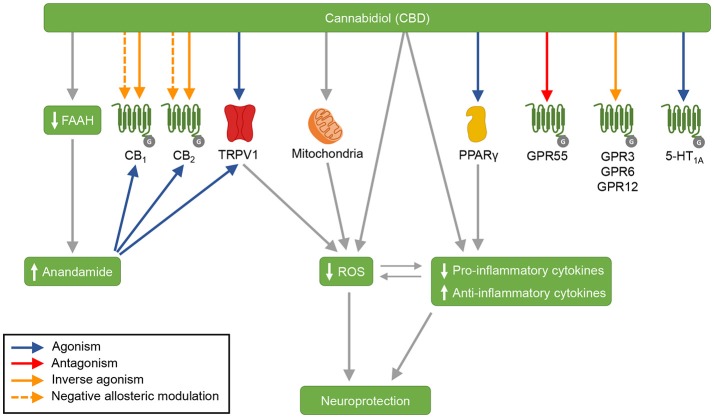
CBD's mechanisms of action. CBD acts as agonist of the receptors TRPV1, PPARγ, and 5-HT_1A_, and as antagonist of the receptor GPR55. CBD is an inverse agonist of the receptors GPR3, GPR6, and GPR12. Moreover, CBD antagonizes the action of CB_1_ and CB_2_ receptors agonists, and is suggested to act as an inverse agonist and a negative allosteric modulator of these receptors. CBD also inhibits FAAH, which results in increased anandamide levels. Anandamide activates CB_1_, CB_2_, and TRPV1 receptors. By acting on mitochondria, CBD increases the activity of mitochondrial complexes. In addition, CBD displays antioxidant and anti-inflammatory effects—that are partially mediated by CBD's actions on TRPV1, mitochondria and PPARγ. 5-HT_1A_, serotonin receptor 1A; CB_1_, cannabinoid receptor type 1; CB_2_, cannabinoid receptor type 2; FAAH, fatty acid amide hydrolase; GPR3, G-protein-coupled receptor 3; GPR6, G-protein-coupled receptor 6; GPR12, G-protein-coupled receptor 12; GPR55, G-protein-coupled receptor 55; PPARγ, peroxisome proliferator-activated receptor gamma; ROS, reactive oxygen species; TRPV1, transient receptor potential vanilloid type 1.

CBD's mechanisms of action are summarized in Figure 1.

## Parkinson's disease (PD)

PD is among the most common neurodegenerative disorders, with a prevalence that increases with age, affecting 1% of the population over 60 years old (Tysnes and Storstein, 2017). The disease is characterized by motor impairment (hypokinesia, tremors, muscle rigidity) and non-motor symptoms (e.g., sleep disturbances, cognitive deficits, anxiety, depression, psychotic symptoms) (Klockgether, 2004).

The pathophysiology of PD is mainly associated with the loss of midbrain dopaminergic neurons in the substantia nigra *pars compacta* (SNpc), with consequent reduced levels of dopamine in the striatum (Dauer and Przedborski, 2003). When the motor symptoms appear, about 60% of dopaminergic neurons is already lost (Dauer and Przedborski, 2003), hindering a possible early diagnosis. The most effective and used treatment for PD is L-DOPA, a precursor of dopamine that promotes an increase in the level of dopamine in the striatum, improving the motor symptoms (Connolly and Lang, 2014). However, after a long-term treatment the effect of L-DOPA can be unstable, presenting fluctuations in symptoms improvement (on / off effect) (Jankovic, 2005; Connolly and Lang, 2014). In addition, involuntary movements (namely L-DOPA-induced dyskinesia) appear in approximately 50% of the patients (Jankovic, 2005).

The first study with CBD on PD patients aimed to verify CBD's effects on the psychotic symptoms. Treatment with CBD for 4 weeks decreased the psychotic symptoms, evaluated by the Brief Psychiatric Rating Scale and the Parkinson Psychosis Questionnaire, without worsening the motor function or inducing adverse effects (Zuardi et al., 2009). Later, in a case series with four PD patients, it was verified that CBD is able to reduce the frequency of the events related to REM sleep behavior disorder (Chagas et al., 2014a). In addition, although not ameliorating PD patients' motor function or their general symptoms score, treatment with CBD for 6 weeks improves PD's patients quality of life (Chagas et al., 2014b). The authors suggest that this effect might be related to CBD's anxiolytic, antidepressant and antipsychotic properties (Chagas et al., 2014b).

Although the studies with patients with PD report beneficial effects of CBD only on the non-motor symptoms, CBD has been shown to prevent and/or reverse increased catalepsy behavior in rodents. When administered before the cataleptic agents haloperidol (antipsychotic drug), L-nitro-N-arginine (non-selective inhibitor of nitric oxide synthase) or WIN 55-212,2 (agonist of cannabinoid receptors), CBD hinders the cataleptic behavior in a dose-dependent manner (Gomes et al., 2013). A possible role of the activation of serotonin receptors 5-HT_1A_ in this action has been proposed, because this effect of CBD is blocked by the pre-treatment with the 5-HT_1A_ antagonist WAY100635 (Gomes et al., 2013). In accordance, Sonego et al. (2016) showed that CBD diminishes the haloperidol-induced catalepsy and c-Fos protein expression in the dorsal striatum, also by a mechanism dependent on 5-HT_1A_ activation. Moreover, CBD prevents the increased catalepsy behavior induced by repeated administration of reserpine (Peres et al., 2016).

In addition, pre-clinical studies in animal models of PD have shown neuroprotective effects of CBD. The unilateral injection of the toxin 6-hydroxydopamine (6-OHDA) into the medial forebrain bundle promotes neurodegeneration of nigrostriatal dopaminergic neurons, being used to model PD (Bové et al., 2005). When inside the cell, the neurotoxin 6-OHDA oxidizes in hydrogen peroxide and paraquinone, causing death mainly of catecolaminergic neurons (Breese and Traylor, 1971; Bové et al., 2005). This neurodegeneration leads to depletion of dopamine and decrease in tyrosine hydroxylase activity in caudate-putamen (Bové et al., 2005; Lastres-Becker et al., 2005). Treatment with CBD during the 2 weeks following 6-OHDA administration prevents these effects (Lastres-Becker et al., 2005). In another study, it was observed that CBD's protective effects after 6-OHDA injury are accompanied by elevation of mRNA levels of the antioxidant enzyme Cu,Zn-superoxide dismutase in substantia nigra (Garcia-Arencibia et al., 2007). The protective effects of CBD in this model do not seem to depend on the activation of CB_1_ receptors (Garcia-Arencibia et al., 2007). In addition to preventing the loss of dopaminergic neurons—assessed by tyrosine hydroxylase immunostaining –, the administration of CBD after 6-OHDA injury attenuates the activation of microglia in substantia nigra (Garcia et al., 2011).

In an *in vitro* study, CBD increased the viability of cells treated with the neurotoxin N-methyl-4-phenylpyrimidine (MPP+), and prevented the MPP+-induced increase in caspase-3 activation and decrease in levels of nerve growth factor (NGF) (Santos et al., 2015). CBD treatment was also able to induce cell differentiation even in the presence of MPP+, an effect that depends on trkA receptors (Santos et al., 2015). MPP+ is a product of oxidation of MPTP that inhibits complex I of the respiratory chain in dopaminergic neurons, causing a rapid neuronal death (Schapira et al., 1990; Meredith et al., 2008).

Data from clinical and pre-clinical studies are summarized in Tables 1, 2, respectively.

**Table 1:** Clinical studies investigating the effects of CBD on movement disorders.

**Disease**	**Main Findings**	**Duration of Treatment**	**Dose of CBD and route of administration**	**Patients characteristics**	**References**
PD	Open-label pilot study. Treatment with CBD for 4 weeks diminished the psychotic symptoms. CBD did not worsen the motor function or induce adverse effects.	4 weeks	150 mg/day of CBD, increasing by 150 mg every week, depending on patients' clinical response. Oral route.	6 PD patients (4 men and 2 women) with psychosis—not controlled with reduction of antiparkinsonian medications—for at least 3 months before the beginning of the study. Patients were in stable doses of anti-PD medication for at least 7 days.	Zuardi et al., 2009
PD	Case series. CBD reduced the frequency of the events related to REM sleep behavior disorder.	6 weeks	75 mg/day (3 patients) or 300 mg/day (1 patient) of CBD. Oral route.	4 PD male patients with REM sleep behavior disorder, with at least two episodes of complex sleep-related behaviors per week.	Chagas et al., 2014a
PD	Exploratory double-blind trial. Treatment with CBD did not improve the motor function or the general symptoms score, but the higher dose (300 mg/kg) improved quality of life.	6 weeks	75 or 300 mg/day of CBD. Oral route.	21 PD patients (15 men and 6 women) in stable doses of anti-PD medication for at least 30 days before the beginning of the study.	Chagas et al., 2014b
HD	Controlled clinical trial (double-blind randomized crossover). Treatment with CBD did not improve the symptoms, but it was not toxic.	6 weeks	10 mg/kg/day of CBD. Oral route.	15 patients (8 men and 7 women) with mild or moderate progression of HD, not taking antipsychotic drugs for at least 2 weeks before the beginning of the study.	Consroe et al., 1991
HD	Double-blind, randomized, cross-over, placebo-controlled, pilot trial. Sativex did not induce severe adverse effects or clinical worsening. However, Sativex did not improve patients' symptoms or promoted molecular changes on biomarkers.	12 weeks	Increasing doses of Sativex (CBD:THC in approximately 1:1 ratio) up to 12 sprays/day. Intranasal route.	25 HD (14 men and 11 women) patients with stable baseline medication for at least 6 weeks before the beginning of the study.	López-Sendón Moreno et al., 2016
HD	Case report of HD patients treated with cannabinoid. Cannabinoids improved UHDRS motor score and dystonia subscore.	6 or 9 months	Sativex: 12 or 7 sprays/day. Intranasal route.	2 male HD patients with complains of severe dystonia. Duration of the disease: 14 and 16 years.	Saft et al., 2018
Dystonic movement disorders	Open label study. Treatment with CBD resulted on 20–50% improvement of the dystonic symptoms. Two patients with simultaneous PD's signs showed worsening of their hypokinesia and/or resting tremor when receiving the higher doses of CBD (over 300 mg/day).	6 weeks	Increasing doses of CBD from 100 to 600 mg/day. Oral route.	5 patients (4 men and 1 woman) with dystonic movements, 2 with simultaneous parkinsonian symptoms.	Consroe et al., 1986
Dystonic movement disorders	Case report. CBD improved the dystonic symptoms without inducing adverse effects.	One administration	CBD 200 mg. Oral route.	2 patients: one woman with idiopathic spasmodic torticollis and one man with generalized torsion dystonia.	Sandyk et al., 1986

*CBD, cannabidiol; HD, Huntington's disease; PD, Parkinson's disease; REM, rapid-eye movement; THC, Δ^9^-tetrahydrocannabinol*.

**Table 2:** Pre-clinical studies investigating the effects of CBD on movement disorders.

**Model**	**Main findings**	**References**
Hamster model of idiopathic paroxysmal dystonia	The higher dose of CBD shows a trend to delay the progression of dystonia.	Richter and Loscher, 2002
PC12 cells expressing mutated huntingtin	CBD and the other three cannabinoid compounds tested—Δ^8^-THC, Δ^9^-THC, and cannabinol—show 51–84% protection against the huntingtin-induced cell death. These protective effects seem to be independent of CB_1_ receptors.	Aiken et al., 2004
Rats lesioned by the toxin 6-OHDA	Treatment with CBD for 2 weeks subsequent to lesion by the toxin 6-OHDA prevents the 6-OHDA-induced depletion of dopamine and decrease in tyrosine hydroxylase activity in caudate-putamen.	Lastres-Becker et al., 2005
Rats lesioned by the toxin 6-OHDA	Treatment with CBD for 2 weeks subsequent to lesion by 6-OHDA prevents the 6-OHDA-induced depletion of dopamine and decrease in tyrosine hydroxylase activity in caudate-putamen. CBD promoted upregulation of mRNA levels for the antioxidant enzyme Cu,Zn-superoxide dismutase. These protective effects do not seem to depend on activation of CB_1_ receptors.	Garcia-Arencibia et al., 2007
Rats treated with 3-nitropropionic acid (3-NP)	Sub-chronic administration of 3-NP reduces GABA contents, levels of mRNA for several markers of striatal GABAergic neurons projections, and the levels of mRNA for the antioxidant enzymes superoxide dismutase-1 (SOD-1) and−2 (SOD-2). CBD reverses or attenuates the 3-NP-induced alterations. CBD's neuroprotective effects are not blocked by antagonists of the CB_1_, TRPV_1_ or A_2A_ receptors.	Sagredo et al., 2007
Rats lesioned by the toxin 6-OHDA	Treatment with CBD for 2 weeks subsequent to lesion by 6-OHDA prevents the 6-OHDA-induced decrease in tyrosine hydroxylase immunostaining, as well as enhanced microglial activation in the substantia nigra.	Garcia et al., 2011
Rats treated with 3-nitropropionic acid (3-NP) or malonate	Sub-chronic administration of 3-NP reduces GABA contents, diminishes the number of Nissl-stained neurons, down-regulates the expression of CB1 receptor and IGF-1, up-regulates the expression of calpain, and reduces the expression of superoxide dismutase-1 (SOD-1). Sativex (CBD and Δ^9^-THC in an approximately 1:1 ratio) attenuates all the 3-NP-induced alterations. This effect is not blocked by antagonists of CB_1_ or CB_2_ receptors. In addition, rats treated with malonate display increased expression of the iNOS gene, reversed by the administration of Sativex.	Sagredo et al., 2011
Rats treated with malonate	Malonate increases edema, decreases the number of surviving cells, enhances the number of degenerating cells, induces strong glial activation, and increases the expression of the inflammatory markers iNOS and IGF-1. Sativex-like combination attenuates all malonate-induced alterations. Sativex effect seems to depend on both CB_1_ and CB_2_ receptors.	Valdeolivas et al., 2012
Mice injected with cataleptic agents	Pre-treatment with CBD dose-dependently attenuates the increase in catalepsy behavior induced by haloperidol, L-nitro-N-arginine or WIN 55,212-2. CBD's anticataleptic effect is prevented by the administration of WAY100635 (antagonist of 5-HT_1A_ receptors).	Gomes et al., 2013
PC12 cells treated with the toxin MPP+	CBD increases cell viability and prevents the MPP+-induced increase in caspase-3 activation and decrease in levels of NGF. CBD treatment also induces cell differentiation even in the presence of MPP+. CBD's effects on neuritogenesis seem to depend on trkA receptors.	Santos et al., 2015
Mice treated with L-DOPA	CBD, when administered with capsazepine, an antagonist of TRPV_1_ receptors, decreases L-DOPA-induced dyskinesia. These effects are blocked by antagonists of CB_1_ and PPARγ receptors. Treatment with capsazepine and CBD also decreases the expression of inflammatory markers (COX-2 and NFkB).	Dos-Santos-Pereira et al., 2016
Rats injected with the cataleptic and dyskinesia-inducing agent reserpine	Repeated administration of reserpine induces catalepsy, hypolocomotion, oral dyskinesia and impairment in the discriminative avoidance memory task. Concomitant treatment with CBD prevents the increase in catalepsy behavior, the oral dyskinesia and the memory deficit.	Peres et al., 2016
Mice injected with the cataleptic agent haloperidol	CBD prevents haloperidol-induced catalepsy and increase in c-Fos protein expression in the dorsolateral striatum. CBD also reverses the increase in catalepsy behavior induced by haloperidol. These CBD effects are prevented by the administration of WAY100635 (antagonist of 5-HT_1A_ receptors). CBD's anticataleptic effect is also observed when CBD is injected into the dorsal striatum.	Sonego et al., 2016
R6/2 mice (transgenic mouse models of HD)	Treatment with Sativex-like combination (from 4th to 12th weeks after birth) attenuated the R6/2 mice increased clasping behavior (that reflects dystonia) and reduced metabolic activity in basal ganglia. Sativex also reversed some of animals' alterations in markers of brain integrity, but not the deterioration in rotarod performance.	Valdeolivas et al., 2017

*Δ^8^-THC, Δ^8^-tetrahydrocannabinol; Δ^9^-THC, Δ^9^-tetrahydrocannabinol; 3-NP, 3-nitropropionic acid; 6-OHDA, 6-hydroxydopamine; CBD, cannabidiol; HD, Huntington's disease; IGF-1, insulin growth factor 1; iNOS, inducible nitric oxide synthase; MPP+, 1-methyl-4-phenylpyridinium; NGF, nerve growth factor; PD, Parkinson's disease; SOD, superoxide dismutase*.

## Huntington's disease (HD)

HD is a fatal progressive neurodegenerative disease characterized by motor dysfunctions, cognitive loss and psychiatric manifestations (McColgan and Tabrizi, 2018). HD is caused by the inclusion of trinucleotides (CAG) in the exons of the huntingtin gene, on chromosome 4 (MacDonald et al., 1993; McColgan and Tabrizi, 2018), and its prevalence is 1–10,000 (McColgan and Tabrizi, 2018). Neurodegeneration in HD affects mainly the striatal region (caudate and putamen) and this neuronal loss is responsible for the motor symptoms (McColgan and Tabrizi, 2018). Cortical degeneration is seen in later stages, and huntingtin inclusions are seen in few cells, but in all patients with HD (Crook and Housman, 2011). The diagnosis of HD is based on motor signs accompanied by genetic evidence, which is positive genetic test for the expansion of the huntingtin gene or family history (Mason and Barker, 2016; McColgan and Tabrizi, 2018).

The pharmacotherapy of HD is still directed toward the symptomatic relief of the disease, i.e., the motor disorders believed to be due to dopaminergic hyperactivity. This treatment is often conducted with typical and atypical antipsychotics, but in some cases the use of dopaminergic agonists is needed (Mason and Barker, 2016; McColgan and Tabrizi, 2018). Indeed, the role of dopamine in HD is not fully elucidated yet. Regarding the cognitive deficits, none of the investigated drugs was able to promote improvements (Mason and Barker, 2016; McColgan and Tabrizi, 2018).

Recently, there has been a growing number of studies aiming to verify the therapeutic potential of cannabinoid compounds in the treatment of HD, mainly because some cannabinoids present hypokinetic characteristics (Lastres-Becker et al., 2002). In a controlled clinical trial, patients with HD were treated with CBD for 6 weeks. There was no significant reduction in the chorea indicators, but no toxicity was observed (Consroe et al., 1991).

The protective effects of CBD and other cannabinoids were also evaluated in a cell culture model of HD, with cells expressing mutated huntingtin. In this model, the induction of huntingtin promotes rapid and extensive cell death (Aiken et al., 2004). CBD and the other three cannabinoid compounds tested—Δ^8^-THC, Δ^9^-THC, and cannabinol—show 51–84% protection against the huntingtin-induced cell death (Aiken et al., 2004). These effects seem to be independent of CB_1_ activation, since absence of CB_1_ receptors has been reported in PC12, the cell line used (Molderings et al., 2002). The authors suggest that the cannabinoids exert this protective effect by antioxidant mechanisms (Aiken et al., 2004).

Regarding studies with animal models, treatment with 3-nitropropionic acid (3-NP), an inhibitor of complex II of the respiratory chain, induces striatal damage—mainly by calpain activation and oxidative injury –, being suggested as relevant to study HD (Brouillet et al., 2005). Sub-chronic administration of 3-NP in rats reduces GABA contents and the levels of mRNA for several markers of striatal GABAergic neurons projections (Sagredo et al., 2007). In addition, 3-NP diminishes the levels of mRNA for the antioxidant enzymes superoxide dismutase-1 (SOD-1) and -2 (SOD-2) (Sagredo et al., 2007). The administration of CBD reverses or attenuates these 3-NP-induced alterations (Sagredo et al., 2007). CBD's neuroprotective effects are not blocked by the administration of antagonists of the CB_1_, TRPV1 or A_2A_ receptors (Sagredo et al., 2007).

More recently, clinical and pre-clinical HD studies started to investigate the effects of Sativex® (CBD in combination with Δ^9^-THC in an approximately 1:1 ratio). In accordance with what previously seen with CBD alone, Sativex administration attenuates all the 3-NP induced neurochemical, histological and molecular alterations (Sagredo et al., 2011). These effects do not seem to be linked to activation of CB_1_ or CB_2_ receptors (Sagredo et al., 2011). Authors also observed a protective effect of Sativex in reducing the increased expression of iNOS gene induced by malonate (Sagredo et al., 2011). Malonate administration leads to striatal damage by apoptosis and inflammatory events related to glial activation, being used as an acute model for HD (Sagredo et al., 2011; Valdeolivas et al., 2012).

In a subsequent study, it was observed that the administration of a Sativex-like combination attenuates all the malonate-induced alterations, namely: increased edema, decreased number of surviving cells, enhanced number of degenerating cells, strong glial activation, and increased expression of inflammatory markers (iNOS and IGF-1) (Valdeolivas et al., 2012). Although the beneficial effects of Sativex on cell survival are blocked by both CB_1_ or CB_2_ antagonists, CB_2_ receptors seem to have a greater role in the protective effect observed (Valdeolivas et al., 2012).

The beneficial effects of Sativex have also been described in the R6/2 mice, a transgenic model of HD. Treatment with a Sativex-like combination, although not reversing animal's deterioration in rotarod performance, attenuates the elevated clasping behavior, that reflects dystonia (Valdeolivas et al., 2017). Moreover, treatment mitigates R6/2 mice reduced metabolic activity in basal ganglia and some of the alterations in markers of brain integrity (Valdeolivas et al., 2017).

In spite of the pre-clinical encouraging results with Sativex, a pilot trial with 25 HD patients treated with Sativex for 12 weeks failed to detect improvement in symptoms or molecular changes on biomarkers (López-Sendón Moreno et al., 2016). Nonetheless, Sativex did not induce severe adverse effects or clinical worsening (López-Sendón Moreno et al., 2016). The authors suggest that future studies, with higher doses and/or longer treatment periods, are in need. More recently, one study described the results of administering cannabinoid drugs to 7 patients (2 of them were treated with Sativex; the others received dronabinol or nabilone, agonists of the cannabinoid receptors): patients displayed improvement on UHDRS motor score and dystonia subscore (Saft et al., 2018).

Tables 1, 2 summarize data from clinical and pre-clinical studies, respectively.

## Other movement disorders

Dystonias are the result of abnormal muscles tone, causing involuntary muscle contraction, and resulting in repetitive movements or abnormal posture (Breakefield et al., 2008). Dystonias can be primary, for instance paroxysmal dyskinesia, or secondary to other conditions or drug use, such as tardive dyskinesia after prolonged treatment with antipsychotic drugs (Breakefield et al., 2008).

Consroe et al. (1986) were the first to evaluate the effects of CBD alone in movement disorders. In this open label study, the five patients with dystonic movement disorders displayed 20–50% improvement of dystonic symptoms when treated with CBD for 6 weeks. Of note, two patients with simultaneous PD's signs showed worsening of their hypokinesia and/or resting tremor when receiving the higher doses of CBD. However, it should be noted that in two more recent studies with PD patients no worsening of motor function was seen (Zuardi et al., 2009; Chagas et al., 2014b). In accordance, Sandyk et al. (1986) reported improvement of dystonic symptoms in two patients—one with idiopathic spasmodic torticollis and one with generalized torsion dystonia—after acute treatment with CBD.

The effects of CBD on dystonic movements were also evaluated in pre-clinical studies. In a hamster model of idiopathic paroxysmal dystonia, the higher dose of CBD showed a trend to delay the progression of dystonia (Richter and Loscher, 2002). In addition, CBD prevents the increase in vacuous chewing movements, i.e., dyskinesia, promoted by repeated administration of reserpine (Peres et al., 2016). CBD's beneficial effects are also seen in L-DOPA-induced dyskinesia in rodents, but only when CBD is administered with capsazepine, an antagonist of TRPV1 receptors (Dos-Santos-Pereira et al., 2016). These effects seem to depend on CB_1_ and PPARγ receptors (Dos-Santos-Pereira et al., 2016). In addition, treatment with capsazepine and CBD decreases the expression of inflammatory markers, reinforcing the suggestion that the anti-inflammatory actions of CBD may be beneficial to the treatment of dyskinesia (Dos-Santos-Pereira et al., 2016).

Moreover, Sativex has been used in the treatment of spasticity in multiple sclerosis. Spasticity is a symptom that affects up to 80% of patients with multiple sclerosis and is associated with poorer quality of life (Flachenecker et al., 2014). A significant portion of patients does not respond to the conventional anti-spasmodic therapies, and some strategies are invasive, posing risks of complications (Flachenecker et al., 2014; Crabtree-Hartman, 2018). Recent data point to Sativex as a valid and well-tolerated therapeutic option. Sativex is able to treat the spasms, improving the quality of life, and displays a low incidence of adverse effects (Giacoppo et al., 2017a).

Data from clinical and pre-clinical studies are summarized in Tables 1, 2, respectively.

## Safety and side effects

One important concern is whether CBD is a safe therapeutic strategy. Several preclinical and clinical reports show that CBD does not alter metabolic and physiological parameters, such as glycemia, prolactin levels, blood pressure, and heart rate. In addition, CBD does not modify hematocrit, leukocyte and erythrocyte counts, and blood levels of bilirubin and creatinine in humans. CBD also does not affect urine osmolarity, pH, albumin levels, and leukocyte and erythrocyte counts. Moreover, *in vitro* studies demonstrate that CBD does not alter embryonic development nor the vitality of non-tumor cell lines. The most reported side effects of CBD are tiredness, diarrhea, and changes on appetite. CBD does not seem to induce tolerance. For a broad review of CBD's side effects, see Bergamaschi et al. (2011) and Iffland and Grotenhermen (2017).

In the context of movement disorders with concomitant cognitive symptoms, as the ones discussed here, it is crucial to evaluate the potential motor and cognitive side effects of CBD. CBD does not induce catalepsy behavior in rodents—being even able to attenuate the effects of several cataleptic agents, as discussed above (El-Alfy et al., 2010; Gomes et al., 2013; Peres et al., 2016; Sonego et al., 2016). In accordance, CBD does not induce extrapyramidal effects in humans (Leweke et al., 2012).

With respect to cognitive effects, studies report that CBD does not impair cognition, being even able to improve it in some conditions. Pre-clinical data show that CBD restores the deficit in the novel object recognition task in mice treated with MK-801 (a protocol used to model schizophrenia) (Gomes et al., 2015), in rats submitted to neonatal iron overload (Fagherazzi et al., 2012), in a transgenic mice model for Alzheimer's disease (Cheng et al., 2014), and in a mice model for cerebral malaria (Campos et al., 2015). CBD also reverses impaired social recognition in a murine model for Alzheimer's disease (Cheng et al., 2014) and restores the deficits in the Morris water maze—a task that evaluates spatial learning—in rodent models for Alzheimer's disease (Martín-Moreno et al., 2011), brain ischemia (Schiavon et al., 2014) and cerebral malaria (Campos et al., 2015). In addition, studies demonstrate that CBD *per se* does not modify animals' performance in cognitive tasks (Osborne et al., 2017; Myers et al., 2018) and does not induce withdrawal after prolonged treatment (Myers et al., 2018). In accordance, in one recent clinical trial using CBD as an adjunctive therapy for schizophrenia, CBD group displayed greater cognitive improvement (assessed by BACS—Brief Assessment of Cognition in Schizophrenia), although it fell short of significance (McGuire et al., 2018). CBD also improves facial emotion recognition in cannabis users (Hindocha et al., 2015).

It is noteworthy that in some cases, particularly concerning multiple sclerosis and HD clinical studies, CBD *per se* does not seem to be beneficial. However, when CBD is administered with Δ^9^-THC in a 1:1 ratio, therapeutic effects are observed. Therefore, it is also important to evaluate the interactions between CBD and Δ^9^-THC as well as the adverse effects of this mixture. Multiple reports point to deleterious effects of Δ^9^-THC on human cognition, mainly on memory and emotional processing (Colizzi and Bhattacharyya, 2017). On the other hand, studies reveal that CBD can counteract Δ^9^-THC detrimental cognitive effects in rodents and monkeys (Wright et al., 2013; Jacobs et al., 2016; Murphy et al., 2017). Nonetheless, this protective effect depends on the doses, on the interval between CBD and Δ^9^-THC administration, as well as on the behavioral paradigm used. In fact, some pre-clinical studies do not observe the protective effect of CBD against the Δ^9^-THC cognitive effects (Wright et al., 2013; Jacobs et al., 2016) or even show that CBD may potentiate them (Hayakawa et al., 2008). Limited clinical evidence indicate that CBD does not worse Δ^9^-THC cognitive effects and, depending on the dose, may protect against them (Colizzi and Bhattacharyya, 2017; Englund et al., 2017; Osborne et al., 2017). Multiple clinical studies with Sativex have not observed motor or cognitive adverse effects (Aragona et al., 2009; Rekand, 2014; López-Sendón Moreno et al., 2016; Russo et al., 2016). Nevertheless, one recent open-label study compared multiple sclerosis patients who continued the treatment with Sativex to those who quitted and reported worse balance and decrease in cognitive performance in the continuers (Castelli et al., 2018). In line with these findings, in an observational study with a large population of Italian patients with multiple sclerosis, cognitive/psychiatric disturbances were seen in 3.9% of the cases (Patti et al., 2016).

## Conclusions

The data reviewed here point to a protective role of CBD in the treatment and/or prevention of some movement disorders. Although the studies are scarce, CBD seems to be effective on treating dystonic movements, both primary and secondary. It is noteworthy that in some cases, particularly concerning multiple sclerosis and HD, the clinical beneficial effects are observed only when CBD is combined with Δ^9^-THC in a 1:1 ratio (Sativex). In fact, these therapeutic effects are probably due to Δ^9^-THC, since they are also seen with other cannabinoid agonists (Curtis et al., 2009; Nielsen et al., 2018; Saft et al., 2018). Nonetheless, CBD is shown to diminish the Δ^9^-THC unwanted effects, such as sedation, memory impairments, and psychosis (Russo and Guy, 2006). Data regarding HD are scarce, but the results of using Sativex in multiple sclerosis are encouraging. Reviews of the clinical use of this compound in the last decade point to effectiveness in the treatment of spasticity as well as improvement in quality of life, with low incidence of adverse effects (Giacoppo et al., 2017a).

In respect to PD, although the pre-clinical studies are promising, the few studies with patients failed to detect improvement of the motor symptoms after treatment with CBD. There is a significant difference between the clinical and pre-clinical PD studies. In animals, the beneficial effects are seen when CBD is administered prior to or immediately after the manipulation that induces the PD-like symptoms. Of note, when treatment with CBD commences 1 week after the lesion with 6-OHDA, the protective effects are not seen (Garcia-Arencibia et al., 2007). These data suggest that CBD's might have a preventive role rather than a therapeutic one in PD. In clinical practice, PD is diagnosed subsequently to the emergence of motor symptoms—that appear up to 10 years after the beginning of neurodegeneration and the onset of non-motor symptoms (Schrag et al., 2015). When the diagnosis occur, approximately 60% of the dopaminergic neurons has already been lost (Dauer and Przedborski, 2003). The fact that in clinical trials CBD is administered only after this substantial progression of the disease might explain the conflicting results. Unfortunately, the early diagnosis of PD remains a challenge, posing difficulty to the implementation of preventive strategies. The development of diagnosis criteria able to detect PD in early stages would probably expand the CBD's applications in this disease.

The molecular mechanisms associated with CBD's improvement of motor disorders are likely multifaceted. Data show that it might depend on CBD's actions on 5-HT_1A_, CB_1_, CB_2_, and/or PPARγ receptors. Moreover, all movement disorders are in some extent linked to oxidative stress and inflammation, and CBD has been reported to display an antioxidant and anti-inflammatory profile, *in vitro* and in animal models for movement abnormalities.

The studies investigating the role of CBD on the treatment of movement disorders are few. Furthermore, differences in the dose and duration of treatment as well as in the stage of the disease (for instance, PD patients are treated only in an advanced stage of the disease) among these studies (shown in detail in Table 1) limit the generalization of the positive effect of CBD and might explain the conflicting results. Notwithstanding, the beneficial neuroprotective profile of CBD added to the preliminary results described here are encouraging. Undoubtedly, future investigations are needed to endorse these initial data and to elucidate the mechanisms involved in the preventive and/or therapeutic potential of CBD on movement disorders.

## Author contributions

All authors listed have made substantial, direct and intellectual contribution to the work, and approved it for publication.

### Conflict of interest statement

JH, and JC are co-inventors (Mechoulam R, JC, Guimaraes FS, AZ, JH, Breuer A) of the patent “Fluorinated CBD compounds, compositions and uses thereof. Pub. No.: WO/2014/108899. International Application No.: PCT/IL2014/050023” Def. US no. Reg. 62193296; 29/07/2015; INPI on 19/08/2015 (BR1120150164927). The University of São Paulo has licensed the patent to Phytecs Pharm (USP Resolution No. 15.1.130002.1.1). The University of São Paulo has an agreement with Prati-Donaduzzi (Toledo, Brazil) to “develop a pharmaceutical product containing synthetic cannabidiol and prove its safety and therapeutic efficacy in the treatment of epilepsy, schizophrenia, Parkinson's disease, and anxiety disorders.” JH and JC have received travel support from and are medical advisors of BSPG-Pharm. The other authors declare that the research was conducted in the absence of any commercial or financial relationships that could be construed as a potential conflict of interest.
